# Prospective Multicenter Study on Early Proximal Tubular Injury in COVID-19–Related Acute Respiratory Distress Syndrome

**DOI:** 10.1016/j.ekir.2024.03.011

**Published:** 2024-03-14

**Authors:** Mickaël Bobot, Xavier Heim, Howard Max, José Boucraut, Pierre Simeone, Claire Stein, Lionel Velly, Nicolas Bruder, Jean-Marie Forel, Sami Hraiech, Christophe Guervilly, Julien Carvelli, Marc Gainnier, Jean-Louis Mège, Sophie Chopinet, Noémie Jourde-Chiche, Laurent Papazian, Stéphane Burtey

**Affiliations:** 1 Centre de Néphrologie et Transplantation Rénale, Hôpital de la Conception, AP-HM, Marseille, France; 2 INSERM, INRAE, C2VN, Aix-Marseille Université, Marseille, France; 3 CERIMED, Aix-Marseille Université, Marseille, France; 4 Laboratoire d’Immunologie, Hôpital de la Conception, AP-HM, France; 5 Département d’Anesthésie-Réanimation, AP-HM, CHU Timone, Aix-Marseille Université, Marseille, France; 6 INT UMR CNRS 7286, Aix-Marseille Université, Marseille, France; 7 CNRS, Institut des Neurosciences de la Timone, UMR7289, Marseille, France; 8 Assistance Publique - Hôpitaux de Marseille, Hôpital Nord, Médecine Intensive Réanimation; Centre d'Etudes et de Recherches sur les Services de Santé et qualité de vie EA 3279, Aix-Marseille Université, France; 9 Service de Réanimation et Surveillance Continue, Hôpital de la Timone, AP-HM, Marseille, France; 10 Service de Chirurgie Générale et Transplantation Hépatique, Hôpital de la Timone, LIIE, CERIMED, Aix-Marseille Université, Marseille, France

**Keywords:** acute kidney injury, ARDS, COVID-19, intensive care, proximal tubular dysfunction, urinary protein electrophoresis

## Abstract

**Introduction:**

During COVID-19, renal impairment is associated with poor prognosis in intensive care unit (ICU). We aimed to assess the existence and incidence of early renal dysfunction and its prognostic value in patients with COVID-19–related acute respiratory distress syndrome (ARDS).

**Methods:**

In this prospective multicenter study, patients aged over 18 years with invasive mechanical ventilation (MV) for ARDS were enrolled in 3 ICUs. Precise evaluation of renal dysfunction markers, including urinary protein electrophoresis (UPE) and quantification, was performed within 24 hours after MV onset.

**Results:**

From March 2020 to December 2021, 135 patients were enrolled as follows: 100 with COVID-19 ARDS and 35 with non–COVID-19 ARDS. UPE found more tubular dysfunction in patients with COVID-19 (68% vs. 21.4%, *P* < 0.0001) and more normal profiles in patients without COVID-19 (65.0% vs. 11.2%, *P* = 0.0003). Patients with COVID-19 significantly displayed early urinary leakage of tubular proteins such as beta-2-microglobulin (ß2m) and free light chains, tended to display acute kidney injury (AKI) more frequently (51.0% vs. 34.3%, *P* = 0.088), had longer MV (20 vs. 9 days, *P* < 0.0001) and longer ICU stay (26 vs. 15 days, *P* < 0.0001). In COVID-19 ARDS, leakage of free lambda light chain was associated with the onset of Kidney Disease: Improving Global Outcomes (KDIGO) ≥2 AKI (odds ratio [OR]: 1.014, 95% confidence interval [CI] 1.003–1.025, *P* = 0.011).

**Conclusion:**

Patients with COVID-19–related ARDS display a proximal tubular dysfunction before the onset of AKI, which predicts AKI. Proximal tubular damage seems an important mechanism of COVID-19–induced nephropathy. Analysis of urinary proteins is a reliable noninvasive tool to assess proximal tubular dysfunction in the ICU.

## Graphical abstract

**Figure:**
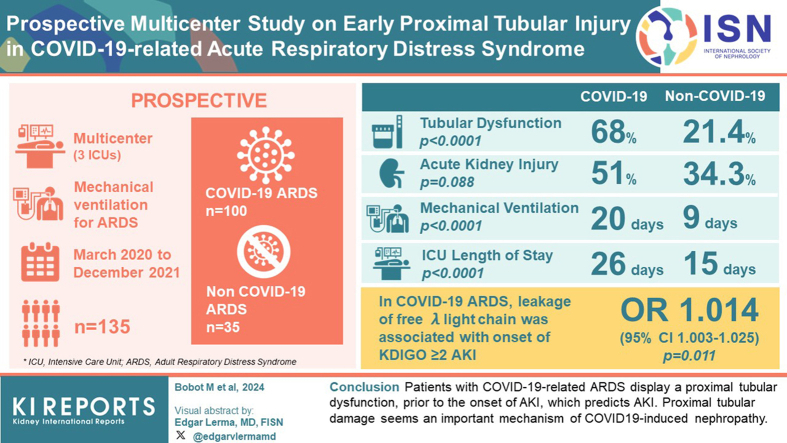

ARDS is the main feature of critically ill patients with COVID-19.^1^, ^2^, ^3^ Renal impairment is the second most frequent organ impairment after lung injury, especially in critically ill patients.^4^, ^5^, ^6^ Several studies found that 20% to 80% of critically ill patients with COVID-19 experienced AKI. Especially AKI was more frequent in patients with who needed MV and developed ARDS.^1^^,^^3^^,^^7^, ^8^, ^9^ Patients hospitalized for COVID-19 developed AKI more frequently than hospitalized patients without COVID-19; with worse outcomes and less recovery of kidney function after AKI.^10^^,^^11^ AKI is of poor prognosis in SARS-CoV-2 infection with a significant association between kidney failure and death or MV.^11^^,^^12^ Previous studies reported various renal subphenotypes, with both tubular and glomerular injuries, where acute tubular injury appeared to be the most frequent.^13^^,^^14^

In hospitalized patients, AKI appears mostly during the first days, the need for renal replacement therapy (RRT) occurred in 5% to 10% of cases^3^^,^^6^ and almost 25% to 50% of patients in ICU, which was strongly associated with mortality, hospital stay, and poor respiratory outcome. The need for RRT occurred more frequently in critically ill patients who underwent MV and those with proteinuria.^9^^,^^11^^,^^15^

For 2 years, several studies about COVID-19 in ICU have been published and renal involvement is no longer in doubt;^13^^,^^16^ however, the understanding of the pathophysiological mechanisms of COVID-19–related AKI is yet to be clarified. Several mechanisms are suspected,^17^ including direct toxicity of the virus in the parenchyma, microvascular thrombosis,^18^^,^^19^ and an imbalanced activation of renin-angiotensin-aldosterone system increasing glomerular permeability.^20^^,^^21^ A proinflammatory state with cytokines storm is probably also involved.^22^^,^^23^

Outside COVID-19, AKI is a frequent complications in ARDS^24^, ^25^, ^26^, ^27^ because there seems to be a crosstalk between lungs and kidneys in critically ill patients,^28^ and it is associated with an overall mortality of 30% to 40% in patients without COVID-19 but with ARDS. However, the urinary profiles of patients with ARDS have been poorly evaluated.

We aimed to perform a detailed analysis of urinary markers of kidney dysfunction in a prospective cohort of critically ill patients in ICU for ARDS due to COVID-19 or other causes, to confirm if SARS-CoV-2 can exert a specific kidney impairment. UPE coupled with quantitative dosage of urinary proteins allow a sensitive and precise analysis of these urinary proteins and to differentiate glomerular and tubular impairment during kidney injury.

The main purpose of our study was to evaluate the incidence of AKI and tubular dysfunction among critically ill patients with COVID-19–related ARDS at the onset of MV, then to specify which part of the kidney parenchyma was involved. The other objectives were to compare the incidence and prognosis of these impairments with patients with non–COVID-19–related ARDS, and to assess the consequences of these kidney impairments on vital and kidney prognosis in ARDS.

## Methods

The *URICOV* study was conducted prospectively in 3 ICUs in the University Hospitals of Marseille, France. Patients aged 18 years or older, with ARDS (PaO2/FiO2 ratio < 300) from COVID-19 or other causes requiring invasive MV were included. Patients with preexisting end-stage kidney disease, in ICU following a lung transplantation of a major cardiothoracic surgery, evident cardiogenic pulmonary edema or moribund (expected death in the 24 hours after ICU admission) were not included. Initial data were collected, and urine and blood were sampled within the first 24 hours after orotracheal intubation. The protocol of the study was registered in *ClinicalTrials.gov (NCT05699889).*

Global initial severity in intensive care patients was assessed by Sepsis-related Organ Failure Assessment and simplified acute physiological score II scores. Severity of ARDS was defined according to the Berlin Definition.^29^ Severity of AKI was defined according to 2012 KDIGO definition,^30^ the follow-up was done at least until hospital discharge, or until the last news. Preexisting chronic kidney disease was defined as a previously known chronic kidney disease in the medical charts or chronic stable estimated glomerular filtration rate < 60 ml/min per 1.73 m^2^.

The following measurements were performed in urinary samples: proteinuria, microalbuminuria, urinary protein-to-creatinine ratio, albumin-to-creatinine ratio, glycosuria, hematuria within the first 24 hours after orotracheal intubation. In 108 patients, UPE was performed. Hematuria was defined as more than 10 red blood cells per μl in urine cytology. Glycosuria was defined as urinary glucose greater than 25 mg/dl.^31^

The diagnosis of COVID-19 was confirmed by reverse transcriptase–polymerase chain reaction performed in the same laboratory of virology on nasopharyngeal swab or tracheal aspiration, using 2 reverse transcriptase–polymerase chain reaction systems, with the LightCycler Multiplex RNA Virus Master kit (Roche Diagnostics, Mannheim, Germany) and a hydrolysis probe.

All biochemical assays were performed with the COBAS 6000 analyzer (Roche Diagnostics, Mannheim, Germany). Serum electrolytes were measured by potentiometry using ion-selective electrodes, creatinine by compensated Jaffe method, and proteinuria by turbidimetry, and microalbuminuria by nephelometry.

The electrophoretic separation of urinary proteins was performed on agarose gel after sodium dodecylsulfate treatment and heating on the Hydrasis apparatus (Sebia, Ivry, France).^32^ Patients were considered to have a normal, tubular, glomerular or mixed proteinuria according to their profile on UPE, qualitatively assessed by a senior immunologist. Tubular profile was defined as a proteinuria mainly composed of low-molecular weight (<68 kDa) proteins (free light chains, retinol-binding protein, alpha-1-microglobulin [α1m], ß2m). Glomerular profile was defined as a proteinuria mainly composed of high-molecular weight proteins (albumin, immunoglobulin G, and transferrin). Mixed profile means the presence of both tubular and glomerular profile in a patient. Normal profile means the absence of detection of urinary protein (Supplementary Figure S1). The urinary determination of α1m, free light chains kappa (FκLC), free light chains lambda (FλLC), and β2m was carried out by immunoturbidimetry on the Optilite apparatus (TheBindingSite, Birmingham, UK, ref. NK036.U, LK016; LK018 and LK48), independently of UPE results. Reference values for urinary α1m are as follows: <7 mg/l, or <13 mg/g of creatinine (in patients aged <50 years) and <20 mg/g of creatinine (in patients aged ≥50 years).^33^ The reference range of urinary β2m was 0 to 0.3 μg/ml.^34^ Free light chains concentrations were measured in using Freelite immunoassays.^35^ Reference values were 0.39 to 15.1 mg/l for FκLC, and 0.81 to 10.1 mg/l for FλLC^36^ (Supplementary Figure S1).

Quantitative values were expressed as means ± SD, except for length of stay expressed in medians (interquartile range) and compared with Student *t*-Test or Mann-Whitney *U* test as appropriate, and Pearson correlation coefficient was calculated. Qualitative values are expressed as *n* (%) and compared with chi-square or Fisher test as appropriated. All tests were 2-tailed. Estimation of survival was done using Kaplan-Meier analysis, and log-rank tests were performed to compare data. Statistical analyses were performed using SAS JMP Pro 14.0 and Graphpad Prism 8.0.1 software. A *P*-value less than 0.05 was considered significant. In multivariate analysis, all variables achieving statistical significance at the 0.10 levels in group comparison were considered for multivariable analysis. A multivariable logistic regression model using backward logistic regression, including all clinically relevant prognostic variables was used to identify prognostic factors of day 28 mortality and KDIGO ≥2 AKI. Collinearity of variables of interest was tested using variance inflation factors.

All procedures were in accordance with the ethical standards of the responsible committee on human experimentation and with the Helsinki Declaration of 1975 revised in 2000 and were registered in Health Data Portal and Commission of Data Protection of Assistance *Publique - Hôpitaux de Marseille* (references *PADS20-105* and *2020-49)* and approved by the ethics committee of the *Société de Réanimation de Langue Française*.^20^, ^21^, ^22^, ^23^, ^24^, ^25^, ^26^, ^27^, ^28^, ^29^, ^30^, ^31^, ^32^, ^33^, ^34^, ^35^ Written information and nonopposition forms were provided to patients or their relatives. The patients and/or their relatives gave their informed consent for the publication of their anonymized data and had the possibility to withdraw their health data. No identifiable individual person’s data is provided in this study.

## Results

### Initial General Characteristics

**Figure 1:**
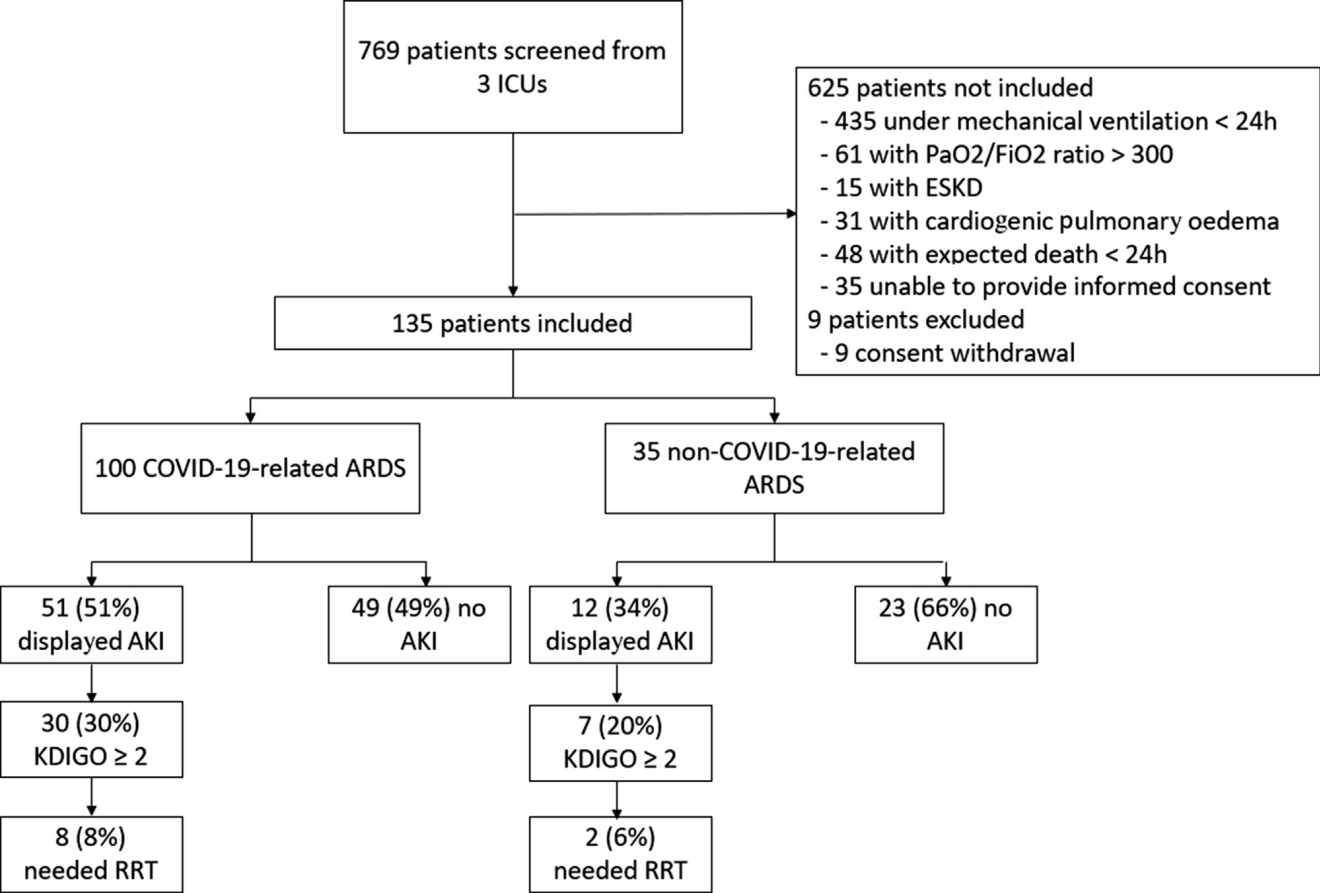
Flow chart of the study. AKI, acute kidney injury; ARDS, acute respiratory distress syndrome; ESKD, end-stage kidney disease; ICU, intensive care unit; KDIGO, Kidney Disease: Improving Global Outcomes; RRT, renal replacement therapy.

From March 21, 2020, to December 22, 2021, 769 patients admitted in ICU for ARDS were screened, of which 135 were included in this cohort, 100 with COVID-19–related ARDS and 35 with non–COVID-19–related ARDS (Figure 1). Patients baseline characteristics, blood and urinary tests performed at inclusion are summarized in Table 1. Patients were mostly male (67%). The mean age was 62 years. In non–COVID-19–related ARDS, the main causes of ARDS were bacterial pneumonia (40%), extrapulmonary ARDS related to septic shock (37%), and inhalation pneumonia (20%). In the COVID-19 group, patients were more frequently obese (46% vs. 7*%, P* = 0.007) whereas patients with non–COVID-19–related ARDS presented with significantly more smoking, chronic lung disease, and active neoplasia (57% vs. 28%, *P* = 0.002; 31% vs. 13%, *P* = 0.014; 14% vs. 1*%, P* = 0.045, respectively). Only 4 patients had chronic kidney disease, and only 2 patients had received nephrotoxic medications (nonsteroidal antiinflammatory drugs).

**Table 1:** Initial characteristics of ARDS patients

Characteristics	All ARDS patients (*N* = 135)	COVID 19 + (*n* = 100)	COVID 19 – (*n* = 35)	*P*-value
Demographic data				
Age (yr)	61.7 ± 11.9	62.1 ± 11.1	60.5 ± 14.2	0.549
Men	91 (67.4)	71 (71.0)	20 (57.1)	0.132
BMI (kg/m^2^)	29.3 ± 6.4	30.4 ± 6.1	26.0 ± 5.9	<0.001
Obesity (BMI ≥30 kg/m^2^)	53 (39.3)	46 (46.0)	7 (20.0)	0.007
Active smoking	48 (35.6)	28 (28.0)	20 (57.1)	0.002
Hypertension	60 (44.4)	47 (47.0)	13 (37.1)	0.312
Atrial fibrillation	10 (7.4)	6 (6.0)	4 (11.4)	0.284
Diabetes	38 (28.1)	33 (33.0)	5 (14.3)	0.034
Dyslipidemia	19 (14.1)	13 (13.0)	6 (17.1)	0.544
Coronary artery disease	16 (11.9)	11 (11.0)	5 (14.3)	0.268
Chronic lung disease	24 (17.8)	13 (13.0)	11 (31.4)	0.014
Chronic kidney disease	4 (3.0)	3 (3.0)	1 (2.9)	0.999
Active neoplasia	6 (4.4)	1 (1.0)	5 (14.3)	0.045
Immunodeficiency	11 (7.4)	8 (8.0)	3 (8.6)	0.999
Solid organ transplant	3 (2.2)	2 (2.0)	1 (2.9)	0.999
Autoimmune disease	3 (2.2)	3 (3.0)	0 (0.0)	0.578
HIV	2 (1.5)	2 (2.0)	0 (0.0)	0.999
Chemotherapy for solid neoplasia	3 (1.5)	1 (1.0)	2 (5.7)	0.165
Treatments before ICU admission				
ACEI	18 (13.4)	12 (12.1)	6 (17.4)	0.454
ARB	24 (17.9)	21 (21.2)	3 (8.6)	0.125
NSAID	2 (1.5)	2 (2.0)	0 (0.0)	0.999
Time between:				
First symptoms to ICU admission (d)	6.7 ± 4.6	7.7 ± 4.3	3.3 ± 3.9	< 0.001
First symptoms to MV (d)	8.5 ± 5.6	9.9 ± 5.3	4.2 ± 4.0	< 0.001
Serum blood tests				
Sodium (mmol/l, 136–145)	137.2 ± 4.7	137.7 ± 4.2	136.0 ± 5.6	0.106
Potassium (mmol/l, 3.40–4.50)	4.1 ± 0.6	4.1 ± 0.5	4.1 ± 0.6	0.716
Bicarbonate (mmol/l, 22–29)	23.1 ± 4.2	22.6 ± 3.1	24.2 ± 6.4	0.189
Albumin (g/l, 35–52)	29.6 ± 5.0	29.4 ± 4.8	30.0 ± 5.6	0.600
Ionized calcium (mmol/l, 1.15–1.33)	1.14 ± 0.10	1.14 ± 0.09	1.14 ± 0.10	0.779
Phosphate (mmol/l, 0.81–1.45)	1.06 ± 0.42	1.02 ± 0.39	1.18 ± 0.49	0.068
Magnesium (mmol/l, 0.66–0.99)	0.90 ± 0.17	0.93 ± 0.16	0.80 ± 0.15	<0.001
Uric acid (μmol/l, 202.3–416.5)	219 ± 118	213 ± 108	234 ± 140	0.469
Glucose (mmol/l, 4.56–6.38)	8.8 ± 3.4	8.9 ± 3.4	8.5 ± 3.6	0.561
Urea (mmol/l, 2.86–8.21)	8.7 ± 5.6	8.7 ± 4.6	8.7 ± 8.0	0.996
Creatinine (μmol/l, 59–104)	90.5 ± 65.6	88.5 ± 62.1	96.5 ± 75.6	0.574
Baseline eGFR (CKD-Epi) (ml/min, >60)	95.2 ± 43.8	94.4 ± 37.2	97.5 ± 59.2	0.775
C-reactive protein (mg/l, <5.0)	172.1 ± 106.5	169.4 ± 100.9	181.2 ± 125.4	0.649
AKI at inclusion	30 (22.2)	18 (18.0)	12 (34.3)	0.046
Stage 1	18 (13.3)	12 (12.0)	6 (17.1)	0.441
Stage 2	7 (5.2)	3 (3.0)	4 (11.4)	0.074
Stage 3	5 (3.7)	3 (3.0)	2 (5.7)	0.604
Urine tests				
Proteinuria (g/l)	0.70 ± 0.74	0.74 ± 0.73	0.57 ± 0.75	0.215
UPCR (g/g, 0–0.5)	0.71 ± 1.19	0.75 ± 1.36	0.58 ± 0.46	0.292
Albuminuria (mg/l)	170 ± 335	173 ± 339	60 ± 408	0.867
UACR (mg/g, 0–30)	177 ± 509	187 ± 571	149 ± 233	0.595
Creatininuria (mmol/l)	9.74 ± 5.1	10.3 ± 5.3	8.0 ± 4.4	0.015
Hematuria	109 (81.3)	84 (84.0)	25 (73.5)	0.176
Glycosuria	94 (75.8)	76 (83.5)	18 (54.5)	<0.001
Urine potassium (mmol/l)	45.3 ± 19.3	44.7. ± 18.5	47.2 ± 21.5	0.539
Urine potassium/creatinine (mmol/g)	55.1 ± 56.6	51.3 ± 60.4	65.8 ± 43.6	0.135
Urinary protein electrophoresis				<0.001
Normal profile	21 (19.4)	9 (11.2)	12 (42.9)	<0.001
Tubular dysfunction	58 (53.7)	52 (65.0)	6 (21.4)	<0.001
Glomerular dysfunction	4 (3.7)	3 (3.7)	1 (3.6)	0.999
Glomerular and tubular dysfunction (mixed profile)	25 (23.1)	16 (20.0)	9 (32.1)	0.190
a-1-microglobulin (mg/l, 0–7)	108.3 ± 84.5	109.2 ± 80.6	105.5 ± 96.7	0.859
a-1-microglobulin/creatinine (mg/g, 0–20)	14.9 ± 15.6	14.0 ± 15.6	16.1 ± 16.0	0.565
b-2-microglobulin (mg/l, 0–0.3)	40.8 ± 52.3	47.6 ± 53.7	22.5 ± 44.4	0.022
b-2-microglobulin/creatinine (mg/g)	5.3 ± 8.2	6.2 ± 8.9	2.9 ± 5.2	0.025
FλLC (mg/l, 0.81–10.10)	83.3 ± 67.1	95.5 ± 65.0	51.1 ± 62.4	0.003
FλLC/creatinine (mg/g)	10.6 ± 11.0	12.1 ± 12.1	6.4 ± 6.0	0.002
FκLC (mg/l, 0.39–15.10)	464.0 ± 429.6	554.1 ± 435.1	225.7 ± 312.1	<0.001
FκLC-to-creatinine (mg/g)	55.1 ± 59.9	65.2 ± 65.4	28.4 ± 28.7	0.0002
Patients’ conditions at inclusion				
SOFA score	6.0 ± 2.9	5.8 ± 2.9	6.5 ± 3.0	0.210
SAPS-II	36.6 ± 11.0	35.5 ± 10.3	39.9 ± 12.5	0.078
Norepinephrin use	41 (31.1)	25 (25.0)	17 (48.6)	0.009
PaO2/FiO2 ratio	103.6 ± 47.4	98.0 ± 40.3	119.1 ± 61.4	0.064
Severe ARDS	80 (59.7)	64 (64.6)	15 (45.7)	0.050
PEEP (cmH20)	12.0 ± 3.1	12.7 ± 2.5	9.7 ± 3.4	<0.001
Tidal volume (ml/kg)	5.47 ± 1.30	5.51 ± 1.24	5.38 ± 1.45	0.682
Static compliance (ml/cmH20)	29.4 ± 9.5	30.0 ± 9.8	27.1 ± 8.5	0.287

ACEI, angiotensin converting enzyme inhibitors; AKI, acute kidney injury; ARB, angiotensin receptor blockers; ARDS, acute respiratory distress syndrome; BMI, body mass index; eGFR, estimated glomerular filtration rate; FκLC, free kappa light chain; FλLC, free lambda light chain; NSAID, nonsteroidal anti-inflammatory drugs; PEEP, positive end expiratory pressure; SAPS-II, simplified acute physiology score II; SOFA, Sepsis-related Organ Failure Assessment; UACR, urine albumin-to-creatinine ratio; UPCR, urine protein-to-creatinine ratio; VitDBP, vitamin D binding-protein.

Qualitative variables are expressed as *n* (%). Quantitative variables are expressed as mean ± SD.

The mean time between onset of symptoms and ICU admission was significantly longer in the COVID-19 group (7.7 ± 4.3 vs. 3.3 ± 3.9, *P* < 0.0001). At inclusion, mean Sepsis-related Organ Failure Assessment score was 6.0 ± 2.9 and mean simplified acute physiological score II score was 36.6 ± 11.0. Simplified acute physiological score II score tended to be higher in the non–COVID-19 group (39.9 ± 12.5 vs. 35.5 ± 10.3, *P* = 0.078) and patients without COVID-19 received more frequently norepinephrine at the time of inclusion (48.6% vs. 25.0%, *P* = 0.009). The mean initial PaO2/FiO2 ratio was not different between the 2 groups (98.0 ± 40.3 in the COVID-19 cohort vs. 119.1 ± 61.4, *P* = 0.604), but ARDS was more frequently severe in the COVID-19 group (64.6% vs. 45.7%, *P* = 0.05). There were no significant differences between the 2 cohorts on MV settings except for the positive end expiratory pressure used, which was higher in the COVID-19 group (12.7 ± 2.5 vs. 9.7 ± 3.4 cmH2O, *P* < 0.0001).

### Initial Biological Characteristics

No difference was observed in routine biochemical serum tests between the 2 groups. The mean creatinine serum level was 90.5 ± 65.6, and 30 patients (22%) displayed AKI among whom 18 had stage 1 AKI. AKI at inclusion was more frequent in the non–COVID-19 group (34% vs. 18%, *P* = 0.046 displayed AKI) (Table 1). Glycosuria was detected in 94 patients (81%) and was more frequent in patients with COVID-19 (83% vs. 54%, *P* = 0.0009), whereas mean serum glucose level was 8.8 ± 3.4 mmol/l in the overall cohort. Hematuria was present in 84.0% of patients with COVID-19 and 73.5% of patients without COVID-19 (*P* = 0.176). Mean urinary protein-to-creatinine ratio and albumin-to-creatinine ratio were comparable between the patients with COVID-19 and those without COVID-19 (0.765 ± 1.36 vs. 0.58 ± 0.46 g/g, *P* = 0.292; and 187 ± 571 vs. 149 ± 233 mg/g, *P* = 0.595, respectively).

**Figure 2:**
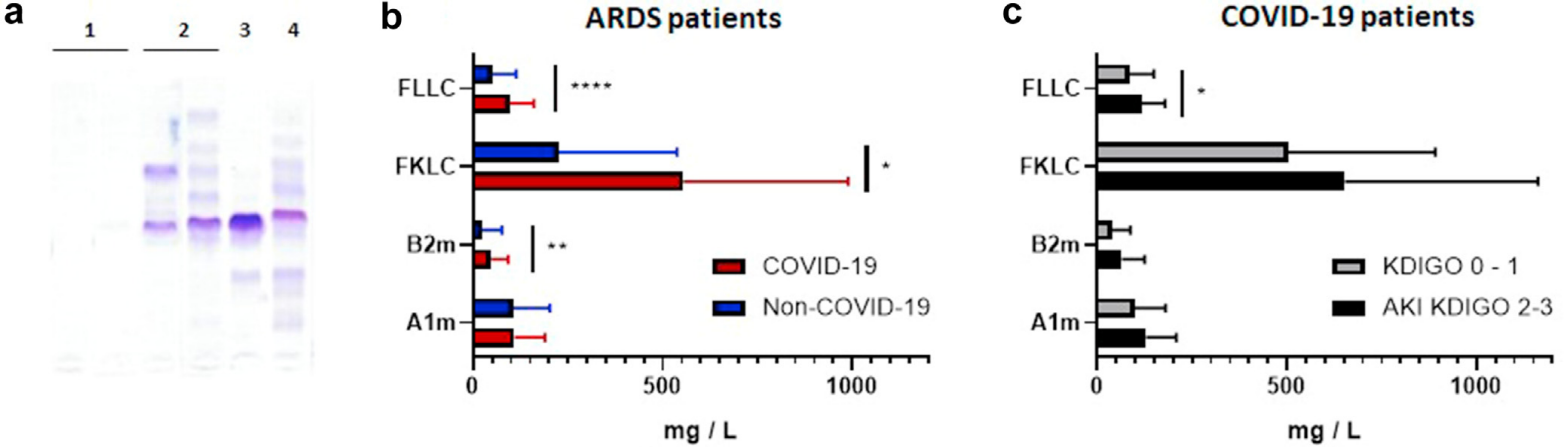
Urinary protein analysis in patients with ARDS. (a) Different urinary profiles in urinary protein electrophoresis: (1) normal profile, (2) tubular profile, (3) glomerular profile, (4) mixed (tubular and glomerular) profile. (b) Urinary protein measurement in patients with ARDS according to the cause of ARDS, (c) urinary protein measurement according to the onset of KDIGO ≥2 AKI in patients with COVID-19–related ARDS. ∗*P* < 0.05, ∗∗*P* < 0.01, ∗∗∗∗*P* < 0.0001. A1m, alpha-1-microglobulin; ARDS, acute respiratory distress syndrome; B2m, beta-2-microglobulin; FKLC, free kappa light chain; FLLC, free lambda light chain; KDIGO, Kidney Disease: Improving Global Outcomes.

UPE was performed in 108 patients and showed a profile of tubular dysfunction in 58 patients (54%). This tubular dysfunction was 3 times more frequent in patients with COVID-19 (65% vs. 21%, *P* < 0.0001) than in patients without COVID-19; and patients without COVID-19 displayed more frequently a normal profile (43% vs. 11%, *P* = 0.003). Only 4 patients displayed a glomerular profile (3 patients with COVID-19 and 1 patient without COVID-19) (Figure 2a).

In the overall population, analysis of urinary proteins showed an important leakage of α1m (108.3 ± 84.5 mg/l), ß2m (40.8 ± 52.3 mg/l), FλLC and FκLC (83.3 ± 67.1 and 464.0 ± 429.6 mg/l, respectively). This low-molecular weight proteins leakage was higher in patients with COVID-19 for ß2m, FλLC, and FκLC (47.6 ± 53.7 vs. 22.5 ± 44.4 mg/l, *P* = 0.022; 95.5 ± 65.0 vs. 51.1 ± 62.4, *P* = 0.003 and 554.1 ± 435.1 vs. 225.7 ± 312.1 mg/l, *P* < 0.0001, respectively) (Table 1, Figure 2b). C-reactive protein levels were significantly correlated to urinary ß2m (r = 0.21, *P* = 0.021) and FλLC (r = 0.234, *P* = 0.045).

### Treatments and Outcomes

Outcomes and patients’ evolution are provided in Table 2. Median follow-up was 48 days. In patients with COVID-19 ARDS, after a median ICU stay of 26 days, 25% died in the ICU, and 51% had experienced AKI (17% stage 3), requiring RRT in 8 patients.

**Table 2:** Evolution and outcomes of the patients in the ICU

Characteristics	All ARDS patients (*N* = 135)	COVID 19 + (*n* = 100)	COVID 19 – (*n* = 35)	*P*-value
Septic shock	73 (54.1)	56 (56.0)	15 (48.6)	0.448
Antibiotics				
Vancomycin	25 (18.5)	20 (20.0)	5 (14.3)	0.454
Aminoglycosides	55 (40.7)	39 (39)	16 (45.7)	0.487
Worst serum urea (mmol/l)	18.5 ± 11.5	19.5 ± 11.4	15.5 ± 11.5	0.080
Worst serum creatinine (μmol/l)	135 ± 114	140 ± 117	121 ± 104	0.363
AKI	63 (46.7)	51 (51.0)	12 (34.3)	0.088
Stage 1	26 (19.3)	21 (21.0)	5 (14.3)	0.386
Stage 2	16 (11.9)	13 (13.0)	3 (8.6)	0.762
Stage 3	21 (15.6)	17 (17.0)	4 (11.4)	0.590
RRT	10 (7.4)	8 (8.0)	2 (5.7)	0.999
RRT duration (d)^a^	11 (2–40)	14 (3–50)	7 (2–12)	0.193
VV-ECMO	21 (15.6)	17 (17.0)	4 (11.4)	0.590
ECMO duration (d)^a^	10 (5–17)	14 (6–20)	7 (4–10)	0.147
Time between first symptoms and ECMO (d)^a^	14 (7–19)	16.5 (11–26)	5 (2–6)	<0.0001
Tracheotomy	45 (33.8)	35 (35.7)	10 (28.6)	0.443
Methylprednisolone (antifibroproliferative regimen)	29 (21.5)	28 (28.0)	1 (2.9)	0.001
iNO	44 (32.6)	37 (37.0)	7 (20)	0.065
iNO duration (d)^a^	6 (3–9)	7 (4–9)	3 (1–4)	0.001
iNO maximum dosage (ppm)	11.9 ± 3.4	11.6 ± 3.2	14 ± 4.2	0.145
Almitrine	16 (11.9)	16 (16.0)	0 (0.0)	0.012
Mortality in ICU	31 (23.0)	25 (25.0)	6 (17.1)	0.341
Mortality at day 28	18 (13.3)	14 (14.0)	4 (11.4)	0.999
Duration of invasive ventilation (d)^a^	17 (8–30)	20 (11–41)	9 (6–22)	<0.0001
ICU stay (d)^a^	21 (13–37)	26 (14–49)	15 (9–25)	<0.0001
Hospital stay (d)^a^	29 (19–48)	31 (19–54)	24 (20–37)	0.012
ICU-free days at day 21	3.2 ± 4.6	2.5 ± 4.1	5.1 ± 5.4	0.016
ICU-free days at day 28	6.4 ± 7.6	5.2 ± 7.1	9.7 ± 8.0	0.005
Follow-up time (d)^a^	48 (29–99)	49 (28–107)	47 (34–98)	0.360

AKI, acute kidney injury; ARDS, acute respiratory distress syndrome; ICU, intensive care unit; iNO, inhaled nitric oxide; RRT, renal replacement therapy; VV-ECMO, veno-venous extracorporeal membrane oxygenation.

Qualitative variables are expressed as *n* (%). Quantitative variables are expressed in mean ± SD.

a Median (interquartiles).

Compared to non–COVID-19 ARDS, patients with COVID-19 tended to experience more AKI (51% vs. 34%, *P* = 0.088). AKI severity in patients with COVID-19 was KDIGO1 in 51% of the patients, KDIGO2 in 21%, and KDIGO3 in 13%; whereas it was 34%, 14%, and 9%, respectively in the patients without COVID-19 (*P* = 0.088). There was no difference in RRT requirement. Fifty-six percent of the patients with COVID-19 displayed septic shock versus 48.6% of the patients without COVID-19 (*P* = 0.445). Patients with COVID-19 required, more frequently, specific ARDS treatments such as methylprednisolone, inhaled nitric oxide (iNO), and almitrine (28% vs. 3%, *P* = 0.001; 37% vs. 20%, *P* = 0.06; 16% vs. 0%, *P* = 0.012, respectively); with longer iNO requirement (7 vs. 3 days, *P* = 0.001), suggesting a more severe or longer severe respiratory condition during the ICU stay. Patients with COVID-19 also had longer invasive ventilation (median, 20 vs. 9 days; *P* < 0.0001) and longer ICU stay (26 vs. 15 days, *P* < 0.0001), but similar mortality at 28 days (14% vs. 11%, *P* = 0.999) and at ICU discharge (25% vs. 17%, *P* = 0.341) as those without covid-19. ICU-free days at day 21 and day 28 were lower in patients with COVID-19 (*P* = 0.016 and *P* = 0.005, respectively) (Table 2).

### Predictive Value of Proximal Tubular Injury on AKI

In COVID-19 ARDS, AKI KDIGO ≥2 was associated with day 28 mortality (30.0% vs. 7.1%, *P* = 0.003), leakage of FλLC (*P* = 0.042), and iNO exposure (*P* = 0.008) (Table 3, Figure 2c). In multivariate analysis, urinary FλLC (OR: 1.014, 95% CI: 1.003–1.025, *P* = 0.011) and iNO exposure (OR: 3.88, 95% CI: 1.127–13.355, *P* = 0.032) remained the only predictive factors associated with AKI KDIGO ≥2. In the overall ARDS population, KDIGO ≥2 AKI was associated with tubular or mixed profile on UPE (93.6% vs. 70.1%, *P* = 0.01), day 28 mortality (27.0% vs. 82%, *P* = 0.004) and ICU-free days at day 28 (3.7 ± 6.9 vs. 7.5 ± 7.6, *P* = 0.008). In multivariate analysis, AKI KDIGO ≥2 was independently associated with higher initial Sepsis-related Organ Failure Assessment score (OR: 1.23, 95%: CI 1.03–1.48, *P* = 0.024), FλLC (OR: 1.012, 95% CI: 1.004–1.020, *P* = 0.004) and lower ICU-free days (OR: 0.85, 95% CI: 0.74–0.98, *P* = 0.025).

**Table 3:** Factors associated with AKI in patients with COVID-19–related ARDS

Variables	KDIGO <2 (*n* = 70)	AKI KDIGO ≥2 (*n* = 30)	*P*-value
Age (yr)	61.2 ± 11.0	64.4 ± 11.2	0.190
Men, *n* (%)	49 (70.0)	22 (73.3)	0.736
Weight (kg)	87.4 ± 17.9	87.1 ± 15.3	0.942
Initial norepinephrine use	14 (20.0)	11 (36.7)	0.078
PaO2/FiO2 ratio	101.3 ± 42.0	90.1 ± 35.3	0.180
PEEP (cmH2O)	13.0 ± 2.7	12.0 ± 2.2	0.065
SOFA score	5.5 ± 2.6	6.5 ± 3.4	0.142
SAPS-II	33.3 ± 9.0	40.1 ± 11.5	0.008
Sodium (mmol/l, 136–145)	137.9 ± 4.1	137.3 ± 4.5	0.533
Potassium (mmol/l, 3.40–4.50)	4.06 ± 0.58	4.26 ± 0.43	0.041
Bicarbonate (mmol/l, 22–29)	23.3 ± 3.0	21.2 ± 2.7	0.001
Ionized calcium (mmol/l, 1.15–1.33)	1.15 ± 0.08	1.11 ± 0.11	0.157
Phosphate (mmol/l, 0.81–1.45)	0.97 ± 0.31	1.13 ± 0.51	0.120
Magnesium (mmol/l, 0.66–0.99)	0.91 ± 0.14	0.96 ± 0.19	0.214
Uric acid (μmol/l, 202.3–416.5)	204.2 ± 91.9	232.7 ± 138.8	0.356
Urea (mmol/l, 2.86–8.21)	7.7 ± 3.3	10.9 ± 6.1	0.012
Creatinine (μmol/l, 59–104)	70.2 ± 24.3	131.0 ± 95.3	0.002
Baseline eGFR (CKD-Epi) (ml/min, >90)	104.7 ± 31.3	70.3 ± 39.3	< 0.001
UPCR (g/g, 0–0.5)	0.61 ± 0.56	1.08 ± 2.33	0.294
UACR (mg/g, 0–30)	130 ± 286	319 ± 946	0.300
Hematuria	59 (84.3)	25 (83.3)	0.905
Glycosuria	53 (81.5)	23 (88.5)	0.421
Urinary protein electrophoresis			0.305
Normal profile	8 (14.5)	1 (4.0)	0.260
Tubular profile	34 (61.8)	18 (72.0)	0.376
Tubular or mixed profile	44 (80.0)	24 (96.0)	0.092
a-1-microglobulin (mg/l, 0–7)	99.8 ± 80.2	128.9 ± 79.6	0.133
a-1-microglobulin/creatinine (mg/g, 0–20)	11.4 ± 12.2	19.5 ± 20.1	0.065
b-2-microglobulin (mg/l, 0–0.3)	39.3 ± 48.5	64.4 ± 60.6	0.085
b-2-microglobulin/creatinine (mg/g)	4.7 ± 7.2	9.3 ± 11.2	0.084
FλLC (mg/l, 0.81–10.10)	84.6 ± 64.3	116.9 ± 62.3	0.042
FλLC/creatinine (mg/g)	10.2 ± 11.9	16.0 ± 11.7	0.048
FκLC (mg/l, 0.39–15.10)	503.8 ± 388.0	652.9 ± 509.3	0.206
FκLC/creatinine (mg/g)	54.9 ± 51.1	85.3 ± 84.5	0.107
Worst urea (mmol/l)	14.2 ± 5.6	31.9 ± 11.9	<0.001
Worst creatinine (mmol/l)	86.9 ± 32.1	265.4 ± 146.5	<0.001
Septic shock	35 (50.0)	21 (70.0)	0.065
Dexamethasone	46 (65.7)	20 (66.7)	0.927
Lopinavir/ritonavir	1 (1.4)	3 (10.0)	0.080
Vancomycin	12 (17.4)	8 (26.7)	0.275
Aminoglycosides	27 (38.6)	12 (40.0)	0.893
iNO	20 (28.6)	17 (56.7)	0.008
iNO duration (d)^a^	8 (2–9)	6 (2–10)	0.821
iNO maximum dosage (ppm)	11.4 ± 2.9	11.8 ± 3.5	0.715
Almitrine	8 (11.4)	8 (26.7)	0.057
VV-ECMO	10 (14.3)	7 (23.3)	0.270
Duration of ECMO support	14.1 ± 8.3	12.0 ± 8.6	0.695
Mortality in ICU	11 (15.7)	14 (47.7)	0.001
Mortality at day 28	5 (7.1)	9 (30.0)	0.003
ICU stay (d)^a^	23 (14–45)	30 (17–56)	0.155
Hospital stay (d)^a^	30 (19–52)	31 (19–59)	0.410
Duration of invasive ventilation (d)^a^	18 (9–39)	23 (13–49)	0.231
ICU-free days at day 21	3.1 ± 4.4	1.3 ± 3.2	0.027
ICU-free days at day 28	6.4 ± 7.3	2.5 ± 5.7	0.005

AKI, acute kidney injury; ECMO, extracorporeal membrane oxygenation; eGFR, estimated glomerular filtration rate; FκLC, free kappa light chain; FλLC, free lambda light chain; ICU, intensive care unit; iNO, inhaled nitric oxide; PEEP, positive end expiratory pressure; SAPS-II, simplified acute physiology score II; SOFA, Sepsis-related Organ Failure Assessment; UACR, urine albumin-to-creatinine ratio; UPCR, urine protein-to-creatinine ratio; VV-ECMO, veno-venous extracorporeal membrane oxygenation.

Qualitative variables are expressed as *n* (%). Quantitative variables are expressed in mean ± SD.

a Median (interquartiles).

### Predictive Factors of Mortality

**Figure 3:**
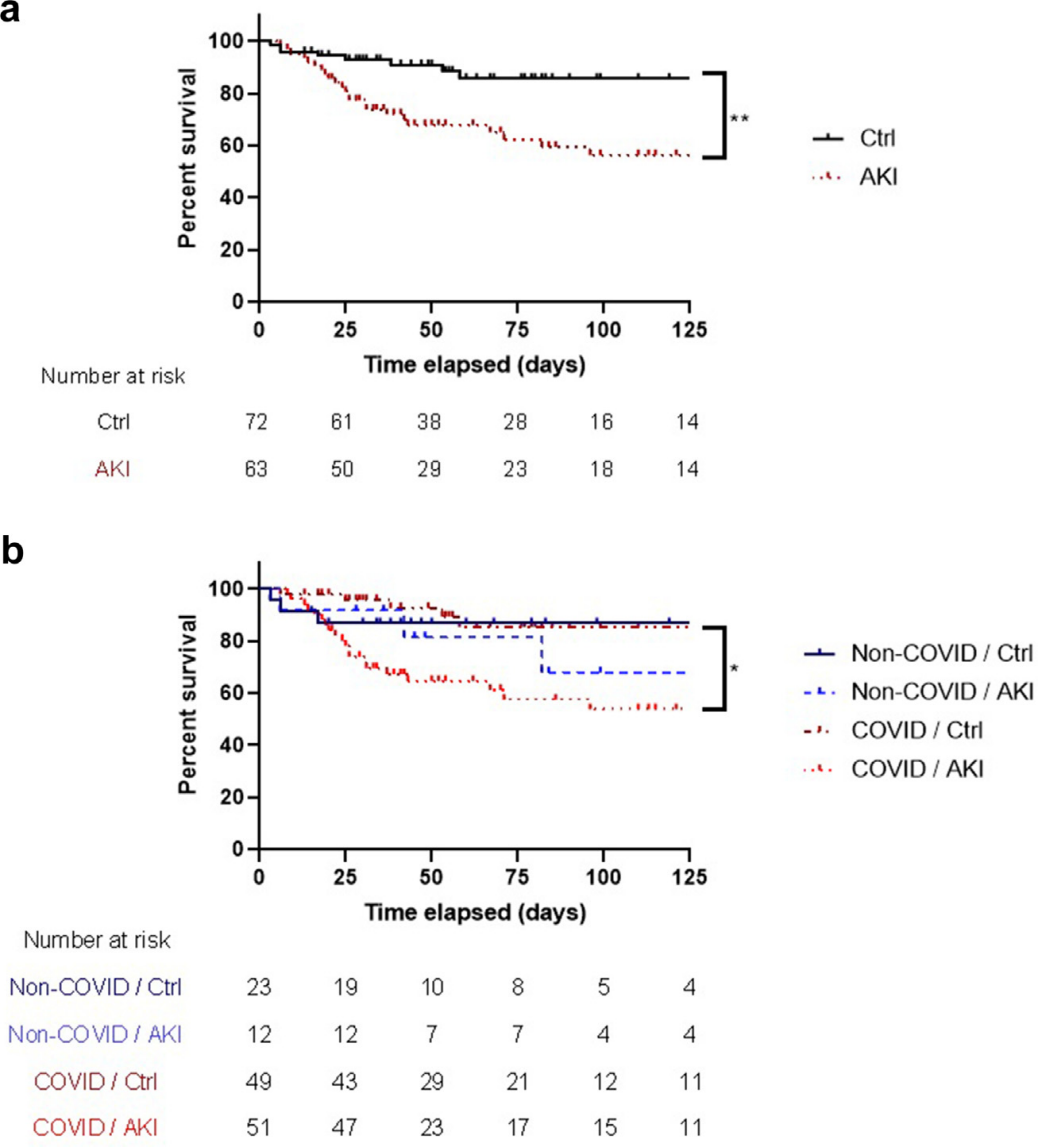
Survival analyses. (a) Survival according to the onset of AKI and the cause of ARDS by Kaplan-Meier plot. (b) Survival according to the onset of AKI in the overall ARDS population by Kaplan-Meier plot. AKI, acute kindey injury; ARDS, acute respiratory distress syndrome; Ctrl, control. ∗*P* < 0.05, ∗∗*P* < 0.01.

In COVID-19 ARDS, patients deceased at day 28 had more frequently experienced AKI (85.7% vs. 45.4%, *P* = 0.008), especially stage 3 (35.7% vs. 14.0%, *P* = 0.04), and had been more frequently exposed to almitrine and iNO (*P* < 0.0001 and *P* = 0.0002, respectively) than those who survived (Table 4). AKI was associated with decreased overall survival among patients with COVID-19–related ARDS (*P* = 0.029, Figure 3a). FλLC leakage was not associated with day 28 mortality (*P* = 0.762). In the overall ARDS population, in multivariate analysis, higher age (OR: 1.12, 95% CI: 1.03–1.22, *P* = 0.008), active neoplasia (OR: 41.49, 95% CI: 4.02–427.86, *P* = 0.002), and iNO exposure (OR: 15.02, 95% CI: 2.83–79.75, *P* = 0.001) were independently associated with mortality. AKI was also associated with decreased overall survival (*P* = 0.002, Figure 3b).

**Table 4:** Factors associated with D28 mortality in COVID-19–related ARDS

Variables	Survived (*n* = 86)	Deceased (*n* = 14)	*P*-value
Age (yr)	60.6 ± 11.0	71.4 ± 6.9	<0.0001
Active neoplasia	1 (1.1)	0 (0.0)	>0.999
Immunodeficiency	7 (8.1)	1 (7.1)	>0.999
SAPS II	34.7 ± 10.3	40.2 ± 9.4	0.073
Initial norepinephrine use	20 (23.3)	5 (35.7)	0.318
AKI	39 (45.4)	12 (85.7)	0.008
AKI severity			0.016
Stage 1	23 (19.7)	3 (16.7)	>0.999
Stage 2	9 (10.5)	4 (28.6)	0.082
Stage 3	12 (14.0)	5 (35.7)	0.044
RRT	6 (7.0)	2 (14.3)	0.311
Urinary protein electrophoresis			0.336
Normal profile	9 (12.9)	0 (0.0)	0.593
Tubular dysfunction	43 (61.4)	9 (90.0)	0.153
Glomerular dysfunction	3 (4.3)	0 (0.0)	0.999
Glomerular and tubular dysfunction (mixed profile)	15 (21.4)	1 (10.0)	0.678
FλLC (mg/l, 0.81–10.10)	96.2 ± 67.9	91.0 ± 45.5	0.762
FλLC/creatinine (mg/g)	12.4 ± 12.8	10.7 ± 6.2	0.528
Dexamethasone	54 (63.8)	12 (85.7)	0.093
iNO	25 (29.1)	12 (85.7)	<0.0001
Almitrine	9 (10.5)	7 (50.0)	0.0002
VV-ECMO	15 (17.4)	2 (14.3)	<0.999

AKI, acute kidney injury; ARDS, acute respiratory distress syndrome; FλLC, free lambda light chain; iNO, inhaled nitric oxide; PEEP, positive end expiratory pressure; RRT, renal replacement therapy; SAPS-II, simplified acute physiology score II; VV-ECMO, veno-venous extracorporeal membrane oxygenation.

Qualitative variables are expressed as *n* (%). Quantitative variables are expressed in mean ± standard deviation.

## Discussion

We show in this study that patients with COVID-19–associated ARDS display features of proximal tubular dysfunction with high prevalence of normoglycemic glycosuria and tubular proteinuria, and that tubular proteinuria is significantly associated with subsequent AKI in ICU. This confirms findings in patients with less severe COVID-19.^15^^,^^37^ The use of UPE as on noninvasive “liquid biopsy” to detect and characterize early kidney injury in patients with ARDS, which had not been described to our knowledge, proved particularly useful.

No kidney biopsy was performed in ICU in this cohort, because of the severe conditions of the patients and frequent anticoagulant treatment. Interestingly, UPE is a noninvasive examination and may be considered as a “liquid biopsy” allowing to differentiate between glomerular and tubular impairment and providing prognostic information, which can find an important application in ICU, where kidney biopsy realization is often difficult. UPE is a highly reproductive semiquantitative orientation tool for proteinuria,^32^ which is well-correlated with single protein determination in urine.^38^ Quantitative dosage of urinary proteins allowed a more sensitive detection of tubular proteins. UPE and urinary protein dosage also have the advantage of being easily available in laboratories compared to other biomarkers of tubular stress or damage such as NGAL or KIM-1. The usual analysis of proteinuria by urinary dipstick, or even by biochemical routine analysis can underestimate the extent of tubular damage.^39^ Indeed, UPE and the direct measurement of the various proteins physiologically reabsorbed by proximal tubular cells revealed an important leak of α1m, β2m, FκLC, FλLC in the urine of most patients with COVID-19 in this cohort reflecting a clear tubular aggression. This is consistent with COVID-19 necropsy series showing mainly acute tubular injury. Our results corroborate the observation of viral particles in proximal tubular cells using electron microscopy and the infection and replication of SARS-CoV-2 in kidney organoids.^14^^,^^40^

We report here a significant prevalence of AKI in patients with COVID-19–related ARDS with 18% of AKI at inclusion and 51% of AKI during the follow-up period. Interestingly, tubular dysfunction was detectable earlier than AKI in most cases and was associated with subsequent AKI. This tubular dysfunction was not related to hemodynamic instability because only 25 patients required norepinephrine at baseline (mainly at low dose due to vasoplegia induced by sedatives). It seems not related to drug toxicity because only 2 patients had received a nephrotoxic drug before admission. Our results are consistent with the work of Kormann *et al.*,^41^ highlighting a high prevalence of incomplete Fanconi syndrome in critically ill patients with COVID-19. The following several causes could contribute to AKI in ARDS: sepsis, dehydratation, fever and digestive symptoms, use of nephrotoxic drugs, hemodynamic instability, MV, hypoxemia causing kidney damage particularly in the medulla and proximal tubules, or changes in renal vascular resistances. Sepsis is highly prevalent in patients with severe COVID-19 and is known to be associated with low-molecular weight proteinuria.^42^ During MV, positive pressure leads to increased central venous pressure, disturbance of venous return, and decrease in cardiac output that lead to inadequate renal perfusion with kidney congestion.^43^^,^^44^ Furthermore, in COVID-19–related ARDS, the systemic release of proinflammatory cytokines such as interleukin, interleukin-6, and tumor necrosis factor-α, caused both by the virus infection and ventilator-induced lung injury, can activate proapoptotic pathway and fibrosis in the kidneys.^28^^,^^43^ We found a significant yet weak association between urinary protein and C-reactive protein levels (due to important variability in C-reactive protein values), suggesting the role of inflammation in kidney injury during severe COVID-19. Interestingly, the exposure to iNO was independently associated with severe AKI in our cohort, which is consistent with recent data on a retrospective cohort of COVID-19–related ARDS,^45^ raising concerns about the nephrotoxicity of iNO in critically ill patients. We deliberately chose to perform UPE at onset of MV to limit these cofounding factors.

In our study, urinary FλLC seems to be the most specific marker of tubular injury because it was independently associated with AKI in COVID-19–related ARDS. We suggest that only FλLC is associated with outcome because urinary free light chains are known to be correlated with systemic inflammation,^46^ unlike α1m or β2m; therefore, free light chains leakage may better reflect kidney inflammation. Under physiologic conditions, FλLC is filtered by the glomerulus less rapidly than FκLC (20%/h vs. 40%/h) because FλLC is dimeric and FκLC is monomeric.^47^ Thus, an increased urinary FλLC may be more specific for proximal tubular damage. Moreover, we found an important variability in urinary FκLC, which can negatively impact the significance of its leakage on outcomes. Thus, urinary FλLC could be an interesting and easily available biomarker of early tubular dysfunction.

AKI is a frequent and one of the most severe features among critically ill patients and can affect one-quarter to more than two-thirds of patients.^48^^,^^49^ It occurs mostly during the first 24 hours after admission in ICU.^50^ There are a few studies evaluating proteinuria in ICU patients; albuminuria seems to be associated with increased mortality and severe renal outcomes, although most of these studies are ancient and included a limited number of patients.^51^

To our knowledge, only 1 small cohort study of 30 patients has focused on tubular proteinuria in critically ill patients with AKI. Tubular proteinuria was associated with higher mortality and need of RRT and was more predictive of RRT than glomerular proteinuria.^52^ However, to date, there is no study evaluating the prognostic interest of UPE on the risk of AKI in and mortality among patients with ARDS.

In 2 large retrospective cohort studies comparing AKI in COVID-19 and influenzae hospitalized patients, AKI occurred significantly more often in patients with COVID-19 (23% vs. 13%), with twice more severe stages (KDIGO ≥2), less renal recovery, and more frequent RRT requirement (13% vs. 2%). Mortality among patients with COVID-19 with AKI appears to be 3-fold higher than in patients with influenza with AKI.^53^^,^^54^

Patients without COVID-19 but with ARDS displayed more AKI at the onset of MV (34% vs. 18%, *P* = 0.046) possibly because of more severe conditions with slightly higher initial simplified acute physiological score II in these patients; whereas patients with COVID-19 would experience more AKI during the ICU stay (51% vs. 34%, *P* = 0.080). Patients with COVID-19 presented mainly with an intense tubular dysfunction, whereas patients without COVID-19 rather displayed a mixed profile with tubular and glomerular dysfunction.

Our study has several limitations. First, there were a few missing data in urine analysis, especially during the first wave of COVID-19. In addition, the population of non–COVID-19 ARDS is small (because of the recruitment during the COVID-19 pandemic) and only 18 patients were deceased at day 28, which limits the statistical power. We do not have access to the dosage of biomarkers such as NGAL, KIM-1, or IGFBP-7, to compare their diagnostic value to tubular protein dosage. Finally, some urinary UPE abnormalities may be explained by patients’ underlying conditions such as diabetes and hypertension. Observed increased glycosuria may also be partly due to diagnosed or undiagnosed diabetes in the context of metabolic syndrome in the COVID-19 group, but no patient was taking sodium-glucose transport protein 2 inhibitors. However, patients with COVID-19 ARDS were more frequently obese and diabetic, 2 conditions that usually result in glomerular rather than tubular damage. We did not evaluate kidney recovery in this study. Gupta *et al.*^12^ found that one-third of the patients remained dependent of RRT on discharge, whereas several studies found that almost 90% of patients recovered from RRT and more than two-thirds of survivors had a full renal recovery.^10^^,^^53^^,^^55^

Our study also has strengths. First, it is a prospective and multicenter study. This is one of the largest cohorts evaluating UPE and specific tubular protein dosages in ICU patients with COVID-19–related ARDS. It covered 5 pandemic waves. This is the first study comparing the impact of specific markers of kidney dysfunction in COVID-19–related ARDS and in non–COVID-19–related ARDS. Our follow-up period was important (median of 48 [29–99] days). To date, only 1 small study evaluated the utility of UPE in AKI before in 31 patients; and found that patients with tubular profile progressed less frequently to chronic kidney disease.^56^ UPE was never evaluated before in the context of ICU and to predict the onset of AKI. We therefore believe our study provides interesting innovative results, by using a rather simple and easily available method allowing a precise evaluation of proteinuria.

These findings open new perspectives. First, studies are needed to confirm our preliminary results in larger ICU cohorts, and in patients with less severe forms of COVID-19, and to evaluate the predictive value of urinary markers of tubular dysfunction for AKI and disease severity. In addition, this work encourages further exploration of urinary protein profiles in patients with other causes of ARDS, such as influenza, to assess if the aggression of proximal tubular cells is specific to SARS-CoV-2 and if it could be of prognostic interest in other conditions at high risk of AKI in the ICU, such as sepsis, cardiac arrest, or cardiac surgery, or in the field of acute nephrology.

## Conclusion

Patients with COVID-19 admitted to the ICU for ARDS display an important proximal tubular dysfunction, before the onset of AKI, which is associated with subsequent AKI. Proximal tubular damage seems an important mechanism of COVID-19–induced nephropathy. Analysis of urinary proteins is a reliable and noninvasive tool that can easily provide important information on early tubular dysfunction and could be useful for the prediction of AKI in the ICU.

## Disclosure

CG reported consulting fees from Xenios FMC outside of the submitted work. All the other authors declared no competing interests.

## Supplementary Material

Supplementary File (PDF) **Figure S1.** Illustrative profiles of urinary protein electrophoresis. From left to right: 1,2,3,4: patients with tubular dysfunction, 5 and 6: patients with tubular dysfunction associated with a glomerular profile.

## Footnotes

Supplementary File (PDF)
**Figure S1.** Illustrative profiles of urinary protein electrophoresis. From left to right: 1,2,3,4: patients with tubular dysfunction, 5 and 6: patients with tubular dysfunction associated with a glomerular profile. α1m, alpha-1-microglobulin; β2m, beta-2-microglobulin; Alb, albumin; App, sample application on gel; ARDS, acute respiratory distress syndrome; FLC, free light chains; RBP, retinol-binding protein; Tr, transferrin.
