# Supplementary material for: Prospective Multicenter Study on Early Proximal Tubular Injury in COVID-19–Related Acute Respiratory Distress Syndrome
Source: Kidney Int Rep. 2024 Mar 14;9(6):1641–53. doi: 10.1016/j.ekir.2024.03.011 (PMC11184390; doi:10.1016/j.ekir.2024.03.011)
Supplement: Supplementary File (PDF) — Figure S1. Illustrative profiles of urinary protein electrophoresis. From left to right: 1,2,3,4: patients with tubular dysfunction, 5 and 6: patients with tubular dysfunction associated with a glomerular profile. [file mmc1.pdf]

Supplementary Figure

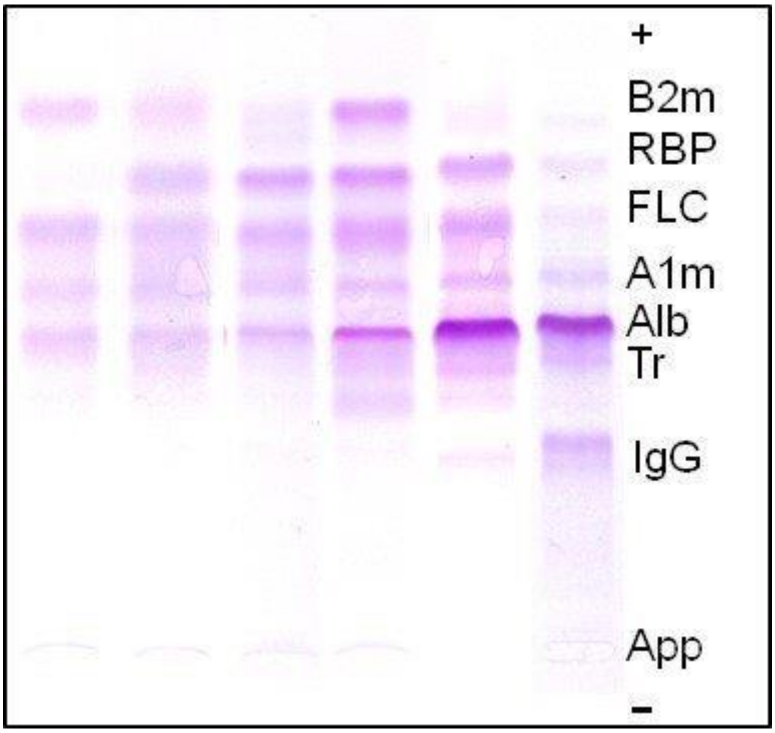

Supplementary Figure S1: Illustrative profiles of urinary protein electrophoresis. From left to right: 1,2,3,4: patients with tubular dysfunction, 5 and 6: patients with tubular dysfunction associated with a glomerular profile. α1m: alpha-1-microglobulin, Alb: Albumin, App: Sample application on gel, ARDS: Acute respiratory distress syndrome, β2m: beta-2-microglobulin, FLC: Free light chains, IgG: immunoglobulin G, RBP: Retinol binding protein, Tr: Transferrin
